# Molecular Subtypes and the Role of TP53 in Diffuse Large B-Cell Lymphoma and Richter Syndrome

**DOI:** 10.3390/cancers16122170

**Published:** 2024-06-07

**Authors:** Ivan Negara, Ciprian Tomuleasa, Sanda Buruiana, Dimitar G. Efremov

**Affiliations:** 1 Molecular Hematology Unit, International Centre for Genetic Engineering and Biotechnology, 34149 Trieste, Italy; 2 Department of Internal Medicine, Hematology, “Nicolae Testemitanu” State University of Medicine and Pharmacy, 2004 Chisinau, Moldova; sanda.buruiana@usmf.md; 3 Department of Hematology, Iuliu Hatieganu University of Medicine and Pharmacy, 400347 Cluj-Napoca, Romania; ciprian.tomuleasa@gmail.com; 4 Macedonian Academy of Sciences and Arts, 1000 Skopje, North Macedonia

**Keywords:** diffuse large b-cell lymphoma, Richter syndrome, chronic lymphocytic leukemia, molecular profiling, genetic classification, TP53, CDKN2A, CDKN2B

## Abstract

**Simple Summary:**

Diffuse large B-cell lymphoma is a heterogeneous entity comprised of several distinct biological subtypes. Multiple molecular studies have attempted to classify this histological subtype and identify potential prognostic factors and treatment targets. Among these, the role of *TP53*, a gene often mutated in many human malignancies, appears particularly relevant. In this review, we summarize the current advances in molecular and genetic subtyping of diffuse large B-cell lymphoma and discuss the pathogenetic and prognostic role of the *TP53* pathway alterations.

**Abstract:**

Diffuse large B-cell lymphoma (DLBCL) is the most common lymphoid malignancy and a heterogeneous entity comprised of several biologically distinct subtypes. Recently, novel genetic classifications of DLBCL have been resolved based on common mutational patterns indicative of distinct pathways of transformation. However, the complicated and costly nature of the novel classifiers has precluded their inclusion into routine practice. In view of this, the status of the *TP53* gene, which is mutated or deleted in 20–30% of the cases, has emerged as an important prognostic factor for DLBCL patients, setting itself apart from other predictors. TP53 genetic lesions are particularly enriched in a genetic subtype of DLBCL that shares genomic features with Richter Syndrome, highlighting the possibility of a subset of DLBCL arising from the transformation of an occult chronic lymphocytic leukemia-like malignancy, such as monoclonal B-cell lymphocytosis. Patients with TP53-mutated DLBCL, including those with Richter Syndrome, have a particularly poor prognosis and display inferior responses to standard chemoimmunotherapy regimens. The data presented in this manuscript argue for the need for improved and more practical risk-stratification models for patients with DLBCL and show the potential for the use of *TP53* mutational status for prognostication and, in prospect, treatment stratification in DLBCL.

## 1. Introduction

Diffuse large B-cell lymphoma (DLBCL) is the most prevalent lymphoid cancer in the world, accounting for around 30–40% of non-Hodgkin lymphomas in adults [1,2]. Although the addition of the anti-CD20 antibody rituximab with the combination of cyclophosphamide, doxorubicin, vincristine, and prednisone (R-CHOP) has improved outcomes in most patient subgroups, up to around 40% of patients do not establish long-term remissions and will succumb to their disease [3,4,5]. In a study comparing R-CHOP to CHOP chemotherapy, young patients had a 6-year event-free survival (EFS) of 74.3% vs. 55.8% and an overall survival (OS) of 90.1% vs. 80% [6], whereas in the elderly, a 10-year progression-free survival (PFS) of 36.5% with R-CHOP (compared to 20% with just CHOP) and a 10-year OS of 43.5% (vs. 27.6%) was reported [7]. However, the treatment of patients who are refractory to first-line therapy with a rituximab-containing regimen still remains an important unmet clinical need [8]. These patients present a dismal life expectancy, with a median OS of less than 12 months [9,10]. In order to predict patient outcomes and pinpoint groups at higher risk of refractory disease, several prognostic models have been developed. Among these is the International Prognostic Index (IPI), which stratifies patients on the basis of easily assessable clinical risk factors [11]. However, while robust and accessible, clinical tools do not fully account for the underlying tumor biology in each risk category. With a wide range of mechanisms, manifestations, and consequences, the disease is remarkably heterogeneous, which is one of the explanations for the lack of success in treatment [1,3,12,13]. At the moment, many novel therapeutic approaches are being developed to target specific pathogenetic pathways related to the underlying tumor biology that could be effective in patients resistant to conventional chemotherapy [14]. Hence, several attempts were made to divide DLBCL into subgroups on the basis of molecular markers in pursuance of better stratification models for outcome prediction and targeted treatment. In this review, we attempt to summarize the recent advances in molecular and genetic classification algorithms and discuss the role of *TP53* in the pathogenesis and prognostication of DLBCL. In addition, we discuss the pathogenic role and prognostic significance of TP53 mutations during the transformation of chronic lymphocytic leukemia (CLL) into DLBCL, a syndrome known as Richter transformation, and discuss similarities between this entity and a genetic subtype of de novo DLBCL also characterized by a high prevalence of TP53 genetic lesions and poor outcomes to treatment with standard chemoimmunotherapy regimens.

## 2. Current Molecular Classifications of DLBCL

### 2.1. Gene Expression Profiling and Cytogenetics

Among the first of the biologic classifications was the cell-of-origin classification (COO), based initially on microarray gene expression profiling (GEP) [15]. DLBCL was thus divided into two major groups, namely germinal center B-cell-like (GCB) and activated B-cell-like (ABC) DLBCL. The latter subtype is characterized by an inferior prognosis, with a 3-year PFS of approximately 40–45% following standard R-CHOP first-line therapy compared to around 75% in GCB-DLBCL [3,16,17].

Comparison of the GEPs of GCB- and ABC-DLBCL with those of normal B cells suggested that these subtypes arise from different stages of B-cell differentiation. In particular, the GCB subtype displays an expression profile similar to that of germinal center B-cells and is characterized by mutations in genes that contribute to maintaining the germinal center phenotype, including *EZH2*, *KMT2D*, *CREBBP*, *EP300*, and *GNA13*, in addition to the common *IGH* and *BCL2* fusion attributed to t(14;18) [3,18]. On the other hand, the cells of the ABC lymphomas are considered to originate from B cells committed to plasmablastic differentiation, although more recent studies suggest that in a fraction of cases, the cell-of-origin could represent memory B-cells undergoing persistent antigen stimulation [19]. These lymphomas are negative for most GC markers and are characterized by genetic alterations affecting the B-cell receptor (BCR) and NF-kB pathways and the B-cell differentiation program, such as mutations in *CD79A/B*, *MYD88*, *CARD11*, *TNFAIP3*, *PIM1*, and *BLIMP1*, and rearrangements of *BCL6* [3,18].

Further adding to the molecular complexity of DLBCL, Monti et al. have identified three additional clusters based on gene expression signatures [20]. These were categorized as “oxidative phosphorylation”, which was enriched in genes related to oxidative phosphorylation and mitochondrial function; “B-cell receptor/proliferation”, characterized by elevated expression levels of cell-cycle regulators and genes involved in the B-cell receptor (BCR) signaling cascade; and “host response”, expressing molecules associated with the T-cell receptor, T/NK-cell activation, and complement cascade.

Hence, the above data suggest that the different subtypes of DLBCL utilize distinct oncogenic pathways and depend on different signaling programs, which could account for the different responses to treatment. In this respect, it is worth noting that most GCB lymphomas are insensitive to the BTK inhibitor ibrutinib, whereas 40% of ABC-DLBCL patients demonstrate clinical responses to treatment with this drug [21], despite evidence that the BCR pathway is frequently activated in both subsets [22,23,24,25]. This difference could be related to the mechanism of BCR activation and the nature of the BCR signal generated in the two subtypes, with chronic active BCR signaling (induced by autoantigen stimulation [26] or autonomous BCR interactions [27]) resulting primarily in the activation of the BTK/NF-kB pathway in ABC DLBCL, and antigen-independent BCR signaling (induced by interactions of mannosylated BCRs with mannose-binding lectins [28] and amplified by deficiency of the phosphatase SHP1 [29]) resulting primarily in the activation of the PI3K/AKT pathway in GCB DLBCL.

As gene expression testing is not readily available in clinical practice, alternative methods have been developed to classify DLBCL. One commonly employed approach is immunohistochemical (IHC) profiling using the Hans algorithm, which classifies DLBCL into GCB and non-GCB types [30]. The non-GCB category includes the ABC cases and those unclassified by gene expression profiling. However, it is now recognized that IHC algorithms cannot correctly distinguish up to around 10–15% of DLBCL cases and are not consistently reported to have prognostic relevance [1,31]. Furthermore, different algorithms assign different weights to various markers, contributing to discrepancies between methods [32]. Hence, incorporating mutational profiles into patient stratification could potentially improve the predictive accuracy of the COO subgrouping.

Indeed, biological classifications are continuously refined with other important markers: cytogenetic studies have shown that *MYC* rearrangements, recognized in around 10–15% of DLBCL (most of the GCB subtype) are frequently associated with *BCL6* and/or *BCL2* translocations and characterize a subset of “extra aggressive” lymphomas, the so-called “double-/triple-hit” (DH/TH) lymphomas [1,33,34,35]. Cases with any combination of these genetic lesions were previously recognized as high-grade B-cell lymphomas in the fourth edition of the WHO classification. However, in the fifth edition, only cases with *MYC* and *BCL2* rearrangements were defined as high-grade B-cell lymphomas because lymphomas with concomitant *BCL6* and *MYC* translocations present a more variable clinical spectrum and were excluded from this category [18]. This distinction is clinically important because high-grade lymphoma patients may benefit more from intensified chemotherapy regimens as opposed to the standard R-CHOP protocol [36,37,38].

Overexpression of MYC and BCL2 in the absence of translocations, termed double-expressor (DE) lymphoma, is much more common; it is associated with the ABC subtype and a seemingly inferior prognosis [31,39,40]. The DE lymphomas arise from a heterogeneous molecular background that may or may not be associated with other types of *MYC* or *BCL2* genetic alterations, such as *BCL2* gains and amplifications [41].

More recent studies have identified additional prognostically unfavorable subgroups characterized by gene expression signatures that are shared with DH/TH lymphomas (double-hit signature, DHITsig) or Burkitt lymphoma (molecular high-grade signature, MHG) but lack *BCL2* and *MYC* rearrangements [42,43,44]. Some of these cases do not overexpress MYC and/or BCL2, suggesting that factors other than MYC and BCL2 overexpression may contribute to the poor prognosis of these patients. 

### 2.2. Genetic Profiling of DLBCL

Recent whole genome and whole exome next-generation sequencing (NGS) studies have allowed for the in-depth characterization of the genomic landscape of DLBCL. Based on these studies, novel classification systems were established, delineating subtypes that more accurately reflect distinct pathogenetic pathways, each associated with different clinical outcomes and potential drug targets [13,45,46,47,48,49,50,51]. However, no consensus has been reached regarding a universal classification. Despite this, considerable overlap was found between the two main classification algorithms, with subgroups defined by similar genomic signatures identified in each of the classification systems. In brief, Staudt and collaborators in two consecutive studies identified a set of seven genetic subtypes that were named after the most common alterations [47,51]. These included the MCD subtype, characterized by MYD88 and CD79B mutations; the EZB subtype, characterized by EZH2 mutations and BCL2 translocations; the BN2 subtype, characterized by BCL6 rearrangements and NOTCH2 mutations; the N1 subtype, characterized by NOTCH1 alterations; the ST2 subtype, characterized by SGK1 and TET2 mutations; the A53 subtype, enriched for TP53 mutations and frequent aneuploidy. The EZB subtype was further stratified to include a prognostically inferior subgroup termed EZB-MYC^+^ (characterized by *MYC* rearrangements, amplifications, and mutations, or the DHIT signature) and a more favorable EZB-MYC^−^ subgroup. In addition, approximately one-third of the cases remained unclassified because they lacked a defining genetic lesion or were genetically composite. 

In the study by Chapuy et al., five genetic clusters termed C1-C5 were described that partially overlap with the previous subtypes [45]. The C1 cluster was associated with BCL6 structural alterations and NOTCH2 mutations, analogous to the BN2 subtype. The C2 cluster was characterized by deletions of 13q14.2/RB1 and CDKN2A/CDKN2B and biallelic TP53 abnormalities, with the latter genetic lesions providing some resemblance with the A53 subtype of DLBCL. The C3 cluster was enriched for BCL2 translocations and mutations in the chromatin modifiers EZH2, CREBBP, and KMT2D and could be aligned with the EZB subtype. The C4 cluster was characterized by SGK1 mutations and H1 linker and core histone alterations, analogous to the ST2 subtype. Finally, the C5 cluster was enriched for MYD88 and CD79B mutations, which are also the defining features of the MCD subtype.

Individual genetic subtypes were shown to be associated with different cell-of-origin DLBCL categories [45,47,51]. Thus, the N1 and MCD/C5 subtypes were strongly correlated with ABC DLBCL; the A53/C2 subtype included a mix of both GCB and ABC DLBCL, while EZB/C3 and ST2/C4 were predominantly associated with the GCB subtype. BN2 tumors were the most diverse, expressing either GCB, ABC, or unclassified GEP signatures, although in the study of Chapuy et al. [45] the majority of the BN2-corresponding C1 DLBCLs were classified as ABC-type tumors. 

**Figure 1:**
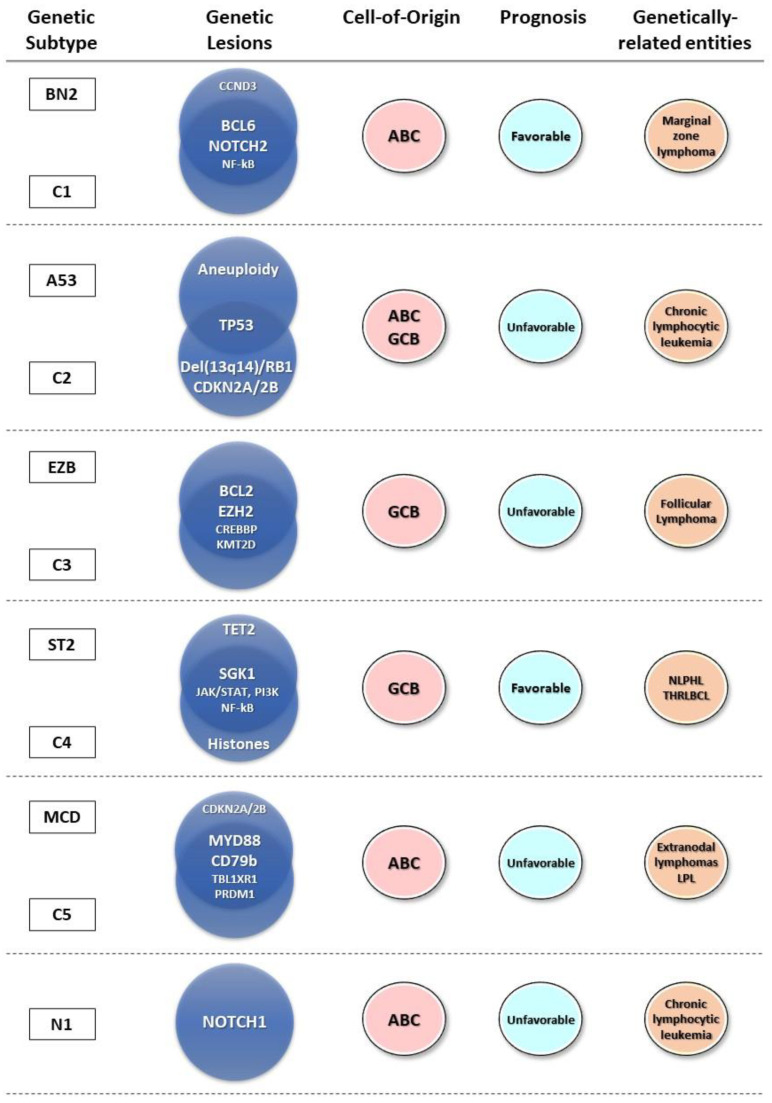
Overlapping features of DLBCL genetic subtypes and related B-cell neoplasms.

A significant mutational overlap was revealed between the genetic subtypes of DLBCL and several other lymphoma entities. The MCD/C5 tumors commonly involve extranodal sites and resemble primary extranodal lymphomas [19,45,51], such as central nervous system, testicular, breast, and female genital tract lymphomas, which are also characterized by a high incidence of co-occurring *MYD88* and *CD79b* mutations [52,53]. These mutations are also frequent in lymphoplasmacytic lymphoma (LPL) [19,54], which can occasionally undergo histological transformation into DLBCL, with 80% of these cases also showing extranodal involvement [55]. With respect to other subtypes, genetic alterations of EZB and C3 have been described in both de novo and transformed follicular lymphoma (FL) [45,51]. Indeed, in the study of Lacy et al., more than a third of DLBCLs assigned to the EZB subtype represented cases of transformed follicular lymphoma or de novo DLBCL with a concurrent diagnosis of FL [48]. Mutational characteristics of the BN2/C1 subtype have been described in marginal zone lymphomas (MZL); however, no histological evidence of pre-existing MZLs was found in the above-mentioned studies, suggesting the possibility of either the transformation of an occult MZL into DLBCL or de novo appearance from a common extrafollicular precursor [46,48,51]. Finally, the ST2 subtype shares genetic similarities with nodular lymphocyte-predominant Hodgkin lymphoma (NLPHL) and T-cell histiocyte-rich large B-cell lymphoma (THRLBCL) (Figure 1) [51].

The different genetic subtypes were also found to display unique gene expression characteristics, suggesting reliance on specific signaling pathways or external stimuli [48,51]. Thus, the MCD subtype was characterized by overexpression of MYC and NF-kB target genes, presumably induced by chronic active BCR signaling, as well as genetic lesions contributing to immune evasion (deletion of CD58 and MHC class I genes), cell cycle deregulation (deletion of CDKN2A) and increased apoptosis resistance (amplification of BCL2). The ST2 subtype was characterized by signatures of PI3K signaling, potentially due to the inactivation of the AKT-family kinase SGK1, and signatures of JAK/STAT signaling, likely promoted by frequent loss-of-function mutations in the negative regulators SOCS1 and DUSP2. The BN2 subtype was characterized by signatures of NOTCH and NF-kB signaling, the latter presumably as a consequence of frequent mutations in components of the NF-kB pathway, such as PRKCB, BCL10, and TNFAIP3. N1 tumors were enriched in NOTCH target genes and expressed a signature of quiescence, whereas EZB-MYC+ tumors were characterized by signatures of proliferation and high MYC activity. Finally, the A53 tumors were characterized by low expression of p53 target genes and frequent deletions or inactivating mutations of the β2-microglobulin gene, providing another mechanism of escape from immune surveillance.

To further explore which pathways are essential for the different genetic subtypes, the group of Staudt performed whole-genome loss-of-function CRISPR/Cas9 in vitro screens in several cell lines belonging to the MCD, BN2 and EZB subtypes [51]. All of the investigated cell lines were found to depend on the expression of a functional BCR, but only the MCD and BN2 cell lines required the expression of BTK and other signaling proteins involved in NF-kB activation [51]. Accordingly, in a follow-up study by the same group, young patients with the MCD subtype had a 100% 3-year OS when treated with R-CHOP in combination with the BTK inhibitor ibrutinib compared to an OS of 69.6% when treated with R-CHOP alone [49].

To further investigate whether the identified overrepresented pathways could guide targeted therapy of the different genetic subtypes of DLBCL, R-CHOP was compared to R-CHOP in combination with a targeted agent in the genetic subtype-guided immunochemotherapy GUIDANCE-01 trial, where a simplified 20-gene algorithm was used to stratify patients in accordance with the classifier of Staudt et al. [56]. The targeted agents included the histone deacetylase (HDAC) inhibitor tucidinostat, which was utilized for the EZB-like subtype because epigenetic alterations are a defining feature of the EZB-type tumors; ibrutinib, which was utilized for the BTK-dependent subtypes MCD-like and BN2-like; and the demethylating agent decitabine, which was utilized for the TP53-mutated subtype, based on its effect in counteracting the immune depletion of the microenvironment associated with TP53-mutated tumors [57,58,59]. Lastly, lenalidomide, an immunomodulatory drug, was utilized for the N1-like subtype and the unclassified patients based on the improved outcome of the lenalidomide + R-CHOP combination in two previous clinical trials of patients with DLBCL and because of the unavailability of a specific targeting agent for patients belonging to these genetic categories [60,61]. Overall, R-CHOP + targeted agent showed a significantly higher overall response rate and complete response rate versus R-CHOP alone (92% vs. 73% and 88% vs. 66%, respectively), as well as a significantly higher 2-year progression-free survival and 2-year overall survival rate (88% vs. 63% and 94% vs. 77%, respectively). 

### 2.3. Genetic Subtypes and Tumor Microenvironment

In addition to the inherent characteristics of the malignant B-cells, the tumor microenvironment (TME) also exhibits a significant influence on the biology and clinical behavior of DLBCL [62]. TME cells (including cytotoxic and helper T-cells, T-regulatory cells, NK cells, monocytes, macrophages, dendritic cells, fibroblasts, endothelial cells, etc.) respond to the presence of neoplastic cells with epigenetic and metabolic changes, as well as changes in gene expression, which often result in either dysfunction of cells that are expected to have an antitumoral role or expansion of cells that become tumor-supporting [63]. Thus, based on the proportions and the phenotypic state of the different T-cell types in the TME, various categories of the lymphoma microenvironment (LME) have been identified [63]. In the study of Kotlov et al., four major lymphoma subtypes were described based on a transcriptomic analysis of 4655 DLBCL samples [64]. These categories included a “germinal center-like” (GC-like) category characterized by the presence of cell types commonly found in germinal centers, a “mesenchymal” (MS) category characterized by the abundance of stromal cells and extracellular matrix signatures, an “inflammatory” (IN) category defined by the presence of inflammatory cells and exhausted and suppressed cytotoxic T cells, and a “depleted” (DP) category characterized by the overall lower presence of a microenvironment-derived signature. Most of the MCD subtype tumors were associated with an IN or DP LME category, whereas BN2 and ST2 tumors showed an enrichment of the MS LME category. In the EZB subtype, the EZB-MYC− tumors were enriched in MS and GC-like LME categories, whereas EZB-MYC+ and A53 tumors had a higher proportion of DP LME categories. In addition, uneven distribution among LME categories was observed for some common mutations and copy number alterations (CNAs), suggesting that the changes in the lymphoma microenvironment are primarily influenced by the genetic factors pertaining to the lymphoma cells themselves. This possibility is further supported by studies showing that specific genetic lesions that define or are over-represented in certain DLBCL genetic subtypes, such as mutations in TP53, NOTCH1, CREBBP, or NFKBIE, can induce changes in the composition or functionality of TME cells that characterize some of the LME categories [57,65,66,67,68,69,70,71,72,73]. Such changes include low infiltration of CD4+ T cells and CD8+ T cells or reduced T-cell cytolytic activity due to immune exhaustion, which are features of the DP and IN category, respectively, and can contribute to the immune escape of the malignant B cells by disrupting T cell-mediated antitumor immune surveillance mechanisms.

### 2.4. Genetic Subtypes and Prognosis

In addition to providing information with respect to the biology of DLBCL, the above-mentioned genetic subtypes also have important prognostic implications. In the study of Chapuy et al., the C1 and C4 tumors were associated with more favorable outcomes, whereas C2, C3, and C5 tumors were prognostically inferior [45]. Accordingly, Wright et al. found the ST2 and BN2 subtypes to be prognostically favorable, especially when compared to the prognostically inferior MCD and N1 DLBCLs, while the EZB, BN2, and A53 tumors displayed an intermediate prognosis [51]. Notably, further stratification of the EZB subtype into EZB-MYC+ and EZB-MYC− categories revealed a significant difference in survival, with patients in the former category experiencing poorer outcomes compared to those in the latter. However, subsequent analysis by Runge et al. [50] reaffirmed only the poor survival of N1 and EZB-MYC+ DLBCL patients and the association of ST2 with a favorable outcome, whereas no difference in outcome was observed between the other genetic subtypes. 

The above studies indicate the potential of genetic classifications in predicting response to treatment and selecting the optimal therapy for DLBCL. However, the novel subtyping approaches may not be easily implemented in clinical practice because they require significant resources and time-consuming bioinformatics analysis of whole-exome or whole-genome sequencing data. To enhance the applicability of the classifications in routine diagnostics, Lacy et al. investigated whether molecular subtypes can be robustly identified using a targeted pan-hematologic malignancy sequencing panel of 293 genes [48]. Despite providing more limited data on translocations and copy number alterations, five subtypes that largely resembled those identified by the LymphGen algorithm in the study of Wright et al. [51] were resolved. These were designated as MYD88, BCL2, NOTCH2, SOCS1/SGK1 and TET2/SGK1. This classification was later modified to include NOTCH1 mutated cases (considering their notable negative prognostic impact) and, similarly to EZB-MYC^+^, a MYC-driven subgroup of the BCL2 subtype [51]. In the absence of data on MYC rearrangements, the latter subgroup was defined only by the presence of mutations in *MYC* codon 57–60, which were previously shown to be strongly associated with *MYC*-rearranged or MHG DLBCL [44,74].

Further efforts to enhance the practicality of the genetic classifications for routine use [75,76] led to the development of a simplified algorithm that relies on the targeted sequencing of 38 genes to identify the seven genetic subtypes based on the LymphGen algorithm [76]. This classifier was tested in a series of 1001 DLBCL patients and showed good concordance with the LymphGen algorithm. However, many cases remain unclassified with either algorithm [27,50,56,77], suggesting that further improvement of the classifiers is required to justify their use in the routine diagnostics setting.

## 3. The *TP53* Pathway and Its Alterations in DLBCL

Mutations and deletions of the *TP53* gene and dysregulation of the p53 pathway play a crucial role in the pathogenesis of many human malignancies, being present in over 60% of cancers; however, they are much less prevalent in hematologic malignancies, accounting for less than 20% of cases [78,79,80]. Despite this, *TP53* alterations have already been established as an unfavorable prognostic factor in a vast spectrum of onco-hematologic disorders, including chronic lymphocytic leukemia/small lymphocytic lymphoma (CLL/SLL), acute lymphoblastic leukemia and various lymphoma subtypes, such as mantle-cell lymphoma, follicular lymphoma, Burkitt lymphoma, and gray-zone lymphoma [79,81,82,83,84,85,86,87]. Importantly, *TP53* alterations are also identified in around 20–30% of de novo DLBCL and are even more prevalent in cases of relapsed/refractory DLBCL [45,47,78,88].

The *TP53* gene is located on the short arm of chromosome 17 (17p13.1), comprising 11 exons and 10 introns, and encoding for p53, a protein considered to be one of the key tumor suppressor genes [89,90]. Human p53 is 393 amino acids long and consists of seven domains, including a transcription-activating domain and a DNA-binding domain (DBD). Activation of p53 occurs in response to cellular stress signals, leading to transactivation or transrepression of multiple target genes involved in various processes, such as apoptosis, senescence, autophagy, and cell cycle arrest. Some of the important downstream events of p53 activation include inhibition of the cyclin-dependent kinases CDK4 and CDK6 through induction of the cyclin-dependent kinase inhibitor CDKN1A (p21), induction of intrinsic apoptosis through transcriptional activation of *Noxa* and *Puma* and through interactions with BCL2, Bcl-xL and BAX, and modulation of the extrinsic apoptotic pathway through induction of TRAIL receptors and *FAS* [78,89,90].

Both the transcription-dependent and transcription-independent activity of p53 is required for tumor suppression, and for both activities, the DBD of p53 plays an important role [91,92]. Mutations occurring in the DBD, encoded by exons 4–9 of the *TP53* gene, are the most common nonsynonymous *TP53* mutations in DLBCL, comprising up to 95% of cases [78,93,94,95,96]. These are commonly linked with deletions of 17p13.1, with prevalence rates ranging from approximately 22% to as high as 50% across different DLBCL populations [45,47,52,93]. *TP53* mutations often lead to a loss of function and are characterized by transcriptional inactivity, as indicated by low p21 protein levels [97]. While mutations in non-DBD regions also occur, a significant difference was observed between alterations in the DBD and non-DBD codons for clinical outcome, with only the former associated with inferior prognosis [94,96,98]. Hence, for routine clinical practice, sequencing of only exons 4–9 and analysis of 17p13.1 deletions is commonly utilized. 

## 4. Prognostic Significance of *TP53* Alterations in DLBCL

Multiple clinical studies in DLBCL have demonstrated both the *TP53* mutations and 17p13.1 deletions to be an independent factor associated with unfavorable prognosis [93,94,98,99,100,101,102,103,104,105,106,107,108,109,110,111,112]. In a large multicenter study investigating the impact of TP53 mutations on the clinical outcome of patients with DLBCL treated with CHOP, TP53 mutations were identified in 102 of the 477 investigated patients and were associated with significantly inferior survival, with a median OS of 1.3 years as compared to 4.5 years in patients without mutations [94]. A subsequent study by the same group showed that the negative prognostic impact of TP53 mutations persisted in patients treated with R-CHOP, with a median OS of 4.4 years in patients with mutated TP53 and 7.9 years in patients with wild-type TP53 [98]. In another large-scale study, TP53 mutations were identified in 24% of the investigated 265 patients and were associated with a decreased 3-year event-free (42% vs. 60%), progression-free (42% vs. 67.5%) and overall survival (50% vs. 76%) as compared to the non-mutated group [100]. It is important to note that the negative prognostic impact of TP53 alterations is retained even when taking into account the recent genetic classifications of DLBCL. Thus, in a large study aiming to establish a simplified algorithm for genetic subtyping of DLBCL, a total of 1001 newly diagnosed patients treated with R-CHOP were evaluated [76]. Therein, survival analysis demonstrated that patients with TP53 mutated tumors displayed a significantly inferior PFS when compared to patients with ST2-like, BN2-like, and EZB-like tumors, indicating that TP53 alterations serve as one of the primary factors for first-line R-CHOP failure and a major contributor to treatment resistance. The findings from these and other studies evaluating the prognostic significance of *TP53* alterations are summarized in Table 1. 

The above data highlight *TP53* abnormalities as a persistent predictor of negative outcomes across various populations of DLBCL patients, regardless of variations in clinical or biological characteristics. In a recent study that employed novel machine learning algorithms combined with data from a targeted transcriptome, only *TP53* mutations persisted as an independent prognostic factor between several multivariate models, unlike overexpression of *MYC* and IRF4, mutations of *MYD88* and *CD79b*, and the cell-of-origin [115]. In a similar fashion, in a study of 117 de novo DLBCL and high-grade lymphoma patients investigating the impact of frequent recurrent mutations on patient outcome, only TP53 mutations were associated with inferior PFS and OS at 2 years [114].

A high prevalence of *TP53* alterations has been correlated with *MYC* translocations in a number of studies and commonly observed in high-grade DH/TH and DHITsig lymphomas [95,106,113,114,116]. In one study, the DHITsig cases with TP53 abnormalities displayed a significantly worse outcome compared to DHITsig patients without *TP53* abnormalities or those with *TP53* mutations lacking the DHITsig, implying the existence of a potential additive effect for these alterations [116]. The adverse prognostic impact of TP53 alterations has also been reported in DE lymphomas, where the loss of *TP53* was associated with chemo-refractory disease and had a synergistic dismal impact on survival [41].

Taking the above into consideration, novel predictive scores are being established that include *TP53* abnormalities in combination with other biological and clinical markers. In one study, PET/CT data from DLBCL patients and a targeted NGS panel consisting of 43 commonly mutated genes were employed to devise a prognostic score [117]. Among the different variables, the pretreatment total metabolic tumor volume, the largest distance between two lesions standardized by body area, and the presence of *TP53* mutations emerged as the most important factors for risk stratification and inferior PFS, displaying better accuracy than current clinical scores. Likewise, a recent algorithm incorporating alterations in *BCL2, TP53*, and IPI showed a better level of accuracy for survival prediction than IPI or R-IPI alone [103].

## 5. *TP53* Alterations in Richter Syndrome

### 5.1. The Interplay between TP53 Alterations, DLBCL and Richter Syndrome

Richter Syndrome (RS) represents the transformation of CLL into an aggressive lymphoma and is currently the most important unmet clinical need in CLL because of the lack of an effective treatment. Richter Syndrome occurs in approximately 5–10% of CLL patients and can present either as a DLBCL variant, which accounts for approximately 90% of the cases, or a Hodgkin lymphoma (HL) variant, which accounts for the remaining cases [118,119,120]. Immunogenetic studies have shown that in more than 80% of the DLBCL cases, the tumor cells are clonally related to the CLL cells, as evidenced by the presence of identical IG gene rearrangements [121]. The remaining cases carry IG gene rearrangements that are different from those of the CLL cells and, therefore, represent de novo lymphomas. This distinction is important because patients with clonally-related and clonally-unrelated Richter Syndrome respond differently to treatment and have a different prognosis. Patients with clonally-related RT are typically resistant to chemoimmunotherapy and have a median overall survival of approximately one year, whereas patients with clonally-unrelated DLBCL generally respond to standard DLBCL treatment and have a similar survival as patients with de novo DLBCL [122].

Early RS genetic studies reported a low frequency or absence of genetic lesions that are frequently detected in de novo DLBCL, such as mutations or copy number alterations in A20, BLIMP1, BCL6, BCL2, CD79B, CARD11, MYD88, CREBBP and EZH2, suggesting that the genomic landscape of RS is significantly different from that of de novo DLBCL and that RS and de novo DLBCL represent distinct disease entities [123,124]. However, the recent genetic classification of DLBCL revealed substantial similarities between clonally related RS and one DLBCL subset. In particular, the most frequent genetic abnormalities in clonally related RS are mutations and deletions of TP53, which are present in 60% to 80% of the cases [123,124,125,126,127]. These genetic lesions frequently co-occur with deletions of the cell cycle inhibitors *CDKN2A* (encoding for p16) and *CDKN2B* (encoding for p15), which are both located on chromosome 9p21 and are deleted in 40–50% of Richter Syndrome tumors but in only 2–6% of untransformed CLL cases [128,129]. Biallelic TP53 and CDKN2A/CDKN2B genetic lesions are also the defining feature of the cluster 2 DLBCL subtype, which is additionally characterized by the frequent presence of the 13q14/RB1 deletion, the most common genetic abnormality of CLL. Moreover, a recent large-scale genome-wide DNA methylation and whole-transcriptome profiling study identified an RS-specific DNA methylation/gene expression signature that was also present in 5.9% of de novo DLBCL tumors [130]. Although no correlation was provided with the DLBCL clusters, it is worth noting that a prominent characteristic of the de novo DLBCL tumors with an RS-specific signature was the frequent expression of the CLL-associated marker CD5, further highlighting similarities between RS and a subset of DLBCL tumors and raising the possibility that some DLBCL tumors may derive from an occult CLL-like malignancy, such as monoclonal B-cell lymphocytosis (MBL). In further support of this possibility, a high incidence of MBL was reported in a series of 165 patients with DLBCL, particularly among patients with ABC DLBCL (28.2%), with a common clonal origin demonstrated in four of the seven investigated cases [131,132].

**Figure 2:**
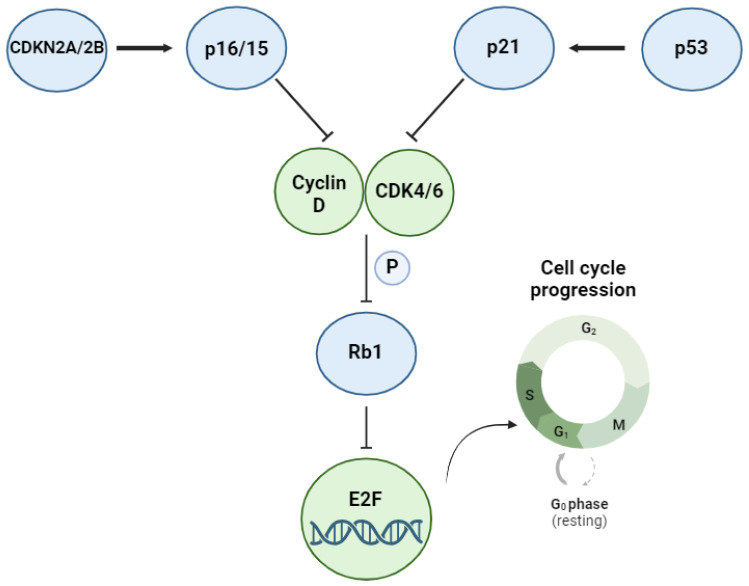
Regulation of cell cycle progression by CDKN2A/2B and TP53. In the presence of mitogenic stimuli, the cyclin-dependent kinases CDK4 and CDK6 phosphorylate RB1, resulting in the release of E2F transcription factors from RB1/E2F complexes. E2F transcription factors then induce the expression of cyclin E, which drives progression from the G1 into the S phase of the cell cycle. CDK4 and CDK6, in turn, are inhibited by the negative cell cycle regulators p15 and p16 (encoded by CDKN2A and CDKN2B) and the p53-regulated cyclin-dependent kinase inhibitor p21.

The frequent co-occurrence of TP53, CDKN2A, and CDKN2B genetic lesions in RS and DLBCL cluster 2 tumors suggests a pathogenic benefit derived from this combination. A possible mechanism of how these genetic lesions could cooperate during lymphomagenesis was revealed in a recent study by our group [133]. Therein, we showed that stimulation of the BCR, which plays a key role in the pathogenesis of CLL, triggers the expression of several positive cell-cycle regulators that promote entry into the G1 phase of the cell cycle but simultaneously induces the expression of p16, p15 and the p53-regulated cyclin-dependent kinase inhibitor p21 (encoded by CDKN1A), thus blocking G1/S phase progression of CLL cells that have been stimulated through the BCR but have not received additional co-stimulatory signals from the tumor microenvironment. This finding raised the possibility that loss of p16, p15, and p21 as a consequence of CDKN2A, CDKN2B, and TP53 genetic lesions, respectively, could contribute to Richter transformation by allowing the malignant B cells to proliferate in the absence of such co-stimulatory growth signals (Figure 2). To further explore this possibility, we used multiplex CRISPR/Cas9 editing to simultaneously target these three genes in autoreactive murine CLL cells derived from the Eμ-TCL1 model. Combined disruption of TP53, CDKN2A, and CDKN2B resulted in the development of rapidly growing RS-like tumors, which contained cells that had acquired the capacity to spontaneously proliferate in vitro. Importantly, the growth of these cells was inhibited by BCR knockout via CRISPR-Cas9 IGHM gene editing or treatment with various BCR inhibitors, suggesting that the proliferation of these cells remains dependent on the expression of a functional BCR. Consistent with these findings, in a subsequent study, we showed that IGHM knockout also delays the growth of xenografted patient-derived Richter Syndrome cells, including cells with combined biallelic TP53, CDKN2A, and CDKN2B genetic lesions [62]. These findings suggest that TP53, CDKN2A, and CDKN2B genetic lesions cooperate with BCR signals in the pathogenesis of Richter Syndrome by reducing the dependence of the malignant B cells on other proliferative microenvironmental signals and raise the possibility that a similar mechanism may be involved in the pathogenesis of cluster two DLBCL.

### 5.2. Prognostic Significance of TP53 Alterations in Richter Syndrome

The current management of Richter Syndrome commonly involves the use of chemoimmunotherapy regimens similar to those utilized in de novo DLBCL, resulting in responses in only 30% to 60% of patients and a median survival of less than 1 year [134,135]. Like in de novo DLBCL, *TP53* mutations and deletions represent an important risk factor for treatment resistance and inferior prognosis in DLBCL-type Richter transformation. In a cohort of RS patients undergoing treatment with chemotherapy and rituximab, those with TP53 alterations had a median OS of only 9.4 months compared to 47.1 months for those without TP53 alterations [122]. The negative prognostic impact of TP53 alterations was more recently corroborated in the study of Parry et al., which identified five RS molecular subtypes by unsupervised non-negative matrix factorization clustering of WES data and showed significantly reduced overall survival of patients with any of the three molecular subtypes characterized by TP53 mutations and/or 17p deletion compared to patients without such abnormalities [127,136]. The above findings suggest that the high prevalence of TP53 genetic lesions may be largely responsible for the chemoresistance and worse outcome of RS patients compared to those with de novo DLBCL, although the impact of CDKN2A/2B genetic lesions also needs to be taken into consideration. In this respect, it is worth noting that homozygous CDKN2A/2B deletions have been associated with venetoclax resistance in RS [137] as well as with a significantly shorter overall survival in de novo DLBCL patients treated with R-CHOP even in the absence of TP53 deletions [138], suggesting that these genetic lesions may independently contribute to treatment failure. 

## 6. Treatment Options for *TP53*-Mutated DLBCL 

Currently, multiple novel management strategies are being investigated for first-line treatment of DLBCL. The combination of R-CHP with polatuzumab vedotin, an antibody-drug conjugate targeting CD79b, demonstrated a marginal improvement in PFS when compared to R-CHOP alone [139]. These results led to its approval for first-line therapy of DLBCL, although it is important to note that this benefit may be restricted to only ABC-type DLBCL [140]. Long-term follow-up of patients from the REMoDL-B trial, which aimed to evaluate the combination of bortezomib with R-CHOP (RB-CHOP) as compared to R-CHOP alone, resulted in an improvement of 5-year OS and PFS in patients with ABC and MHG DLBCL [141]. As already mentioned, inhibition of BTK with ibrutinib demonstrated efficacy in a subset of patients with ABC-DLBCL belonging to the BCR-dependent MCD and N1 subtypes [50]. In addition, potential synergy was shown between several targeted agents in the Smart Start study, with a combination of rituximab, lenalidomide, and ibrutinib (RLI) followed by CHOP or EPOCH chemotherapy showing a CR rate of 94.5% and a 2-year PFS of 91.3% in non-GCB DLBCL patients [142].

Despite this, limited data exist regarding the efficacy of novel agents in TP53-mutated DLBCL. Neither intensified chemotherapy regimens nor radiation therapy, either delivered locally or as a radiolabeled monoclonal antibody, overcome the negative prognostic impact of *TP53* mutations [104,106,143,144,145], emphasizing the need for novel targeted therapies. However, this is made difficult due to the nature of *TP53* abnormalities, as the desired outcome for novel agents would be to restore the function of the mutated or absent p53 protein, which poses a significant challenge [146]. In acute myeloid leukemia and myelodysplastic syndrome, where *TP53* mutations also delineate a subtype with particularly poor outcomes, eprenetapopt (APR-246), a novel drug that acts by restoring normal folding and function of mutant p53, showed promise in phase I and phase II clinical trials [147,148,149]. Although no clinical trials with this drug are currently ongoing in lymphoma, it is worth noting that eprenetapopt in combination with idasnutlin, an inhibitor of the TP53 negative regulator MDM2, showed synergistic activity against TP53-impaired DLBCL cell lines in vitro [150]. In addition, a recent study demonstrated that arsenic trioxide is also able to stabilize the structure and rescue the transcriptional activity of a wide range of p53 mutants [151], suggesting that it could represent another drug with potential activity against TP53-mutated cancers, including DLBCL.

The role of BCR inhibitors in the treatment of the RS-related C2 subset has not yet been evaluated and appears potentially worthwhile in view of our studies showing a detrimental effect of BCR disruption on the growth of murine RS models and RS patient-derived xenograft cells with combined TP53 and CDKN2A/2B genetic lesions [62,133], as well as small-scale clinical trials demonstrating responses in 40–75% of RS patients treated with a BTK inhibitor [152,153,154,155]. Moreover, BCR inhibitors have been shown to sensitize DLBCL cells to the BCL2 inhibitor venetoclax [29], which is another agent that has demonstrated activity in RS, particularly in combination with chemoimmunotherapy [156,157]. The presence of genetic alterations affecting the BCR-dependent NF-kB pathway (such as mutations in *CD79B*, *MALT1*, *TRAF6*, *TNIP1*, *NFKBIZ*, and *TNFAIP3*) in around 45% of A53 DLBCL cases further suggests that this pathway could represent a potential therapeutic target in a subset of DLBCL cases with TP53 mutations [51].

Dysregulation and depletion of the TME is a well-documented feature of *TP53*-mutated DLBCL [57,58,63,64,158]. Consistent with the potential role of *TP53* in shaping the immune microenvironment of the tumor, *TP53* alterations in lymphomas are associated with reduced activity and migration of CAR-T cells and serve as a potent predictor of anti-CD19 CAR-T failure [158]. Decitabine, a DNA methyltransferase inhibitor, was previously shown to induce tumor microenvironment remodulation and potentially enhance the T cell-mediated antitumor immune response [59,159]. In *TP53*-mutated DLBCL, epigenetic alterations leading to depletion of the tumor immune microenvironment were found to be alleviated with the inclusion of decitabine in vitro and in patient-derived xenograft models, and an upregulation of the interferon program and increased T-cell activation was demonstrated in *TP53*-mutated patients treated with decitabine [58]. Accordingly, in a study aiming to evaluate the addition of targeted agents to first-line R-CHOP treatment based on the genetic subtype of DLBCL, patients with the TP53-mutated subtype treated with R-CHOP combined with decitabine (DR-CHOP) demonstrated a better response rate as compared to those treated with R-CHOP alone [56], indicating the potential of targeting the immune microenvironment in DLBCL with TP53 abnormalities. Still, the effectiveness of immune-based strategies for this DLBCL subgroup remains to be validated.

## 7. Conclusions

In recent years, a number of large-scale genomic studies revealed the existence of several genetic subtypes of DLBCL, each presenting with a different prognostic impact and reliant on distinct signaling pathways. These discoveries have improved the existing risk-stratification models of DLBCL, and the subsequent clinical studies have showcased the potential benefit for patient outcomes by incorporating subtype-dependent targeted agents—like BTK-inhibitors, epigenetic modulating agents, and immunomodulatory drugs—into the standard first-line R-CHOP chemoimmunotherapy regimen. In addition, a different, less discussed aspect of the DLBCL genetic subtypes is their manifestation with mutational spectrums that may overlap with various other lymphoid malignancies. Thus, similar genetic characteristics were demonstrated between MCD/C5 and primary extranodal lymphomas, EZB/C3 and FL, BN2/C1 and MZL, and ST2-type DLBCL with NLPHL and THRLBCL. Further supporting the notion of existing shared pathogenetic mechanisms, significant resemblances are discernable between the DLBCL-type Richter transformation and the C2 subset of DLBCL, with abnormalities in *TP53* and the cell-cycle regulators *CDKN2A/2B* commonly co-occurring in both entities, thus highlighting the potentially overlooked prospect of DLBCL arising from the transformation of an indolent B-cell neoplasm, such as occult lymphoma or monoclonal B-cell lymphocytosis.

However, a significant number of cases remain unclassified with the novel algorithms, and the adoption of these methods into clinical settings may encounter obstacles due to their time-intensive nature and substantial resource requirements, emphasizing the need for more practical stratification models. With respect to this, the data presented in this manuscript demonstrates that *TP53* mutations and deletions are associated with inferior survival and resistance to first-line therapy across clinically and biologically diverse populations of DLBCL and high-grade lymphoma patients. Thus, even when taking into account the various genetic classifiers, *TP53* abnormalities remain a highly prevalent, persistent, and accessible predictor of unfavorable patient outcomes in DLBCL. 

*TP53* mutations and deletions are also likely major contributors to the treatment resistance and particularly poor prognosis of DLBCL-type Richter transformation. Because of the functional consequences induced by these abnormalities, neither the traditional nor the more intensive chemoimmunotherapy regimens elicit positive treatment responses, and currently no established clinical procedures are available to improve patient outcomes in either de novo DLBCL or DLBCL-type RS with *TP53* alterations. However, recent advances in understanding the pathogenetic pathways linked to TP53 abnormalities have led to the emergence of potential novel agents, and both the BCR pathway and the immune microenvironment of the tumor may hold promise as therapeutic targets for these patients. Based on the above, we argue that additional prospective trials aimed at further investigation of these therapeutic targets in *TP53*-deficient DLBCL and RS are warranted in order to improve the current treatment strategies and, ultimately, outcomes for these patients.

## Figures and Tables

**Table 1:** Prevalence and prognostic impact of *TP53* alterations across various studies.

Number of Patients	Prevalence of *TP53* Alterations	Complete Response Rate (*TP53*^mut^ vs. *TP53*^wt^) ^a^	Survival (*TP53*^mut^ vs. *TP53*^wt^) ^a^	Reference
125	9%	36% vs. 80%	5-year OS 34% vs. 83% 5-year FFS 24% vs. 72%	[104]
196	14.8%	Poor in *TP53*^mut^	Poor in *TP53*^mut^	[113]
74	16.2%	58% vs. 82%	5-year OS 42% vs. 69%	[93]
506	21.9%	60% vs. 80%	5-year OS 48% vs. 66% 5-year PFS 46% vs. 64%	[98]
69	23%	N/A	2-year OS 62% vs. 88% 2-year PFS 58% vs. 80%	[106]
265	23.8%	62% vs. 80%	3-year OS 50% vs. 76% 3-year PFS 42% vs. 68%	[100]
170	24.1%	N/A	Median OS: 12.55 months vs. not reached	[95]
81	29%	50% vs. 87% ^b^	Poor OS, TTP in *TP53*^mut^	[105]
191	30.9%	N/A	5-year OS 53% vs. 66% 5-year PFS 30% vs. 36%	[103]
117	36%	55% vs. 77%	2-year OS 70% vs. 99% 2-year PFS 57% vs. 77%	[114]
477	21.4%	57% vs. 69% ^c^	5-year OS 19% vs. 45%	[94]
102	22%	27% vs. 76%	5-year OS 16% vs. 64%	[111]
69	23%	69% vs. 83% ^d^	6-year OS 44% vs. 79%	[109]

FFS, failure-free survival; OS, overall survival; PFS, progression-free survival; TTP, time to progression. ^a^ *p*-values for all comparisons are less than 0.05, unless specified otherwise, ^b^ Also includes patients that relapsed within 12 months since treatment initiation, ^c^ *p* = 0.089, ^d^ *p* = 0.16.
